# Supplementary material for: Rice bran oil emulgel as a pork back fat alternate for semi-dried fish sausage
Source: PLoS One. 2021 Apr 22;16(4):e0250512. doi: 10.1371/journal.pone.0250512 (PMC8062034; doi:10.1371/journal.pone.0250512)
Supplement: Attachment — Submitted filename: Response to Reviewers.docx [file pone.0250512.s002.docx]

**Response to Reviewers**

All points raised by the editor and reviewer were carefully addressed and answered point-by-point. A revision was made in highlighted red fonts. The revised manuscript was carefully prepared to meet PLOS ONE's style requirements.

ACADEMIC EDITOR: According to the comments of reviewer 2 and my opinion as an academic editor, again the authors are suggested to revise the manuscript to improve the missing discussion highlighting the explanation of mechanisms, emulgel structuration, texture, and SEM pictures with available references in literature to support the results.

**Ans:** The manuscript was revised accordingly in which the mechanisms, emulgel structuration, texture, and SEM pictures were discussed.

Reviewer #2: Authors have addressed all minor comments I have made in previously version. Nevertheless, the major comment regarding the mechanism and lack of explanation on the emulgel structuration is a issue that was not answered. Although authors have said they have done their best to explain texture and SEM pictures for both reviewers questions, however they have not even tried to improve. There are a lot of references available to support these missing discussion. I believe only shows the changing in physical properties is not enough for a scientific paper. The explanations of all whys have always to followed the changes.

**Ans:** Thank you very much for your invaluable suggestions. A revision was made carefully as suggested. The emulgel structuration, texture, and SEM pictures were discussed intensively (See L293-L262). The references were also updated.

1. Gani A, Benjakul S. Impact of virgin coconut oil nanoemulsion on properties of croaker surimi gel. Food Hydrocoll. 2018;82:34-44.
2. Chandra MV, Shamasundar BA. Texture profile analysis and functional properties of gelatin from the skin of three species of fresh water fish. Int. J. Food Prop. 2015;18:572-584.
3. Ashara KC, Paun JS, Soniwala MM, Chavada JR, Mori NM. Micro-emulsion based emulgel: a novel topical drug delivery system. Asian Pac. J. Trop. Dis. 2014;4:S27-S32.
4. Cubides YTP, Eklund PR, Foegeding EA. Casein as a modifier of whey protein isolate gel: sensory, texture and rheological properties. J. Food Sci. 2019;84:3399-3410.
5. Zhang T, Xu X, Ji L, Li Z, Wang Y, Xue Y, Xue C. Phase behaviors involved in surimi gel system: Effects of phase separation on gelation of myofibrillar protein and kappa-carrageenan. Food Res. Int. 2017;100:361-368.
6. Ayadi MA, Kechaou A, Makni I, Attia H. Influence of carrageenan addition on turkey meat sausages properties. J. Food Eng. 2009;93:278-283.
7. Sun XD, Holley RA. Factors influencing gel formation by myofibrillar proteins in muscle foods. Compr. Rev. Food Sci. Food Saf. 2011;10:33-51.
8. Liu J, Zhu K, Ye T, Wan S, Wang Y, Wang D, Wang C. Influence of konjac glucomannan on gelling properties and water state in egg white protein gel. Food Res. Int. 2013;51:437-443.
9. Poklukar K, Čandek-Potokar M, Batorek Lukač N, Tomažin U, Škrlep M. Lipid deposition and metabolism in local and modern pig breeds: A review. Animals. 2020;10:424.
10. de Figueiredo Furtado G, Mantovani RA, Consoli L, Hubinger MD, da Cunha RL. Structural and emulsifying properties of sodium caseinate and lactoferrin influenced by ultrasound process. Food Hydrocoll. 2017;63:178-188.
11. Leon AM, Medina WT, Park DJ, Aguilera JM. Properties of microparticles from a whey protein isolate/alginate emulsion gel. Food Sci. Technol. Int. 2018;24:414-423.
12. Ortiz J, Aguilera JM. Effect of kappa-carrageenan on the gelation of horse mackerel (*T. murphyi*) raw paste surimi-type. Food Sci. Technol. Int. 2004;10:223-232.

Moreover, authors forgot to highlight the changes as they have said they did. What makes really hard to follow the changes.

**Ans:** In the revision 1, there were 2 files including ‘manuscript’ and ‘manuscript with track changes’. We highlighted what we have changed in the red fonts. In this revision (R2), the revision was made in the highlighted red fonts as well.
